# Development of a Topical Resveratrol Formulation for Commercial Applications Using Dendrimer Nanotechnology

**DOI:** 10.3390/molecules22010137

**Published:** 2017-01-14

**Authors:** Tyler Pentek, Eric Newenhouse, Brennin O’Brien, Abhay Singh Chauhan

**Affiliations:** 1 School of Pharmacy, Concordia University Wisconsin, 12800 N. Lake Shore Drive, Mequon, WI 53097, USA; Tyler.pentek@cuw.edu (T.P.); eric.newenhouse@cuw.edu (E.N.); brennin.obrien@cuw.edu (B.O.); 2 School of Pharmacy, Medical College of Wisconsin, Milwaukee, 8701 W. Watertown Plank Road, Milwaukee, WI 53226, USA

**Keywords:** resveratrol, dendrimer, PAMAM, solubility, stability, transdermal, topical, cream, antiaging, safe

## Abstract

Resveratrol (RSV) is well known for its anti-oxidant and anti-aging properties. However, resveratrol is insoluble in water and has stability issues. Recently, efforts were placed to prepare a resveratrol-based advanced anti-aging topical product but it contains harsh organic solvents and oils that could be harmful to the human body and the environment. Hence, we propose the use of a multifunctional dendrimer to solve the solubility and stability issues of resveratrol. A dendrimer-resveratrol complex was prepared, optimized and tested for solubility enhancement, stability in solution and cream dosage forms. We have also developed a high performance liquid chromatography method to measure the resveratrol within the final product. PAMAM dendrimers increased the solubility and stability of resveratrol in water and semisolid dosage forms. Therefore, this product would be water based ‘green’ formulation devoid of harsh organic solvents and oils and can be safely applied to the skin. Additionally, we have shown that the dendrimer helped to increase overall RSV loading and skin penetration of resveratrol. The dendrimer-RSV formulation was successfully scaled up towards commercialization. Dendrimer with RSV has led to an innovation in anti-aging cream and solutions that could be commercially marketed. Dendrimer-RSV complex could also be added to other product forms for additional purposes and applications.

## 1. Introduction

**Figure 1:**
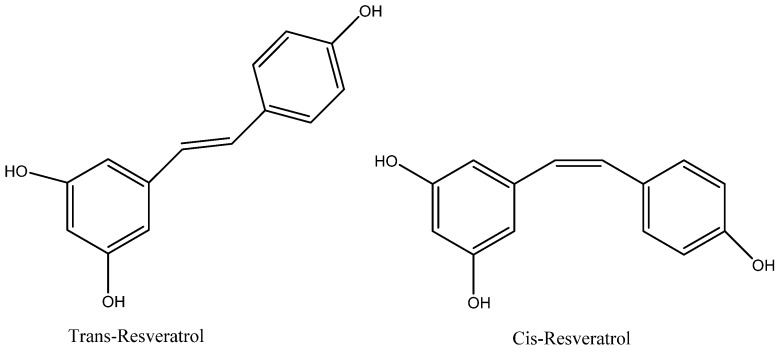
Resveratrol isomers.

A wide variety of plant food sources, particularly berries, are a source for stilbenes and natural phenolic compounds [1,2]. Resveratrol (3,4′,5-trihydroxystilbene, RSV) is one of these stilbene compounds that has two known isoforms—*trans*-resveratrol and *cis*-resveratrol (Figure 1)—of which *trans*-resveratrol is determined to be the more stable compound of the two [1,2]. The isomerization of the *trans*-isoform to the *cis* isoform is caused by two factors: UV light and high pH. However, the *cis* to *trans* isomerization is influenced by visible light, high temperatures and low pH [1,2]. RSV was first discovered in 1940 in the roots of white hellebore (*Veratrum grandiflorum*) [1,2]. RSV is commonly found within the skin of red grapes, mulberries, peanuts, pines and *Polygonum cuspidatum* weed root extracts [1,2]. In these plants RSV acts as an antifungal agent and protects the plant from various infections. Grapes are often infected with *Botryis cinerea*, which increases the levels of RSV within the adjacent grapes [2]. RSV is produced within these plants by fungi, stress, injury, infection or by UV radiation [3,4]. Additionally, environmental stress (UV light and heavy metals) have an impact on the overall increased levels of RSV in various plants [3,4].

Current uses for RSV include treatment for inhibiting cancer, slowing aging, cardio-vascular disease, antiviral treatments, inflammation, platelet aggregation and other applications [2,5]. RSV as an anticancer treatment has been linked with regulatory pathways that have both growth and death attributes [5]. Additional evidence suggests that RSV helps to maintain genome stability [5]. Other studies have signified that RSV is a cancer chemopreventative treatment, which has demonstrated the ability to inhibit tumor growth of a wide variety [5]. One drawback of RSV given orally is that the drug undergoes very rapid metabolism within the body, which overall limits the bioavailability of the molecule at the site of action [5]. Reports have now suggested that even low doses of RSV that are consumed via the common diet may have a beneficial effect in reducing cardio-vascular disease [5]. Additional evidence has demonstrated that RSV can increase the potency of a selection of antiviral drugs in the treatment of HIV [5]. Other studies have indicated that resveratrol can have an anti-inflammatory effect by suppressing tissue factors and cytokines within vasculature cells [6]. The antioxidant effects of RSV come from the scavenging of intracellular reactive oxygen species (ROS), which maintains the overall concentration within biological systems in the body. Resveratrol studies have indicated that even with oral doses of up to 5 grams of *trans*-resveratrol have not shown any major adverse effects in humans [7].

The use of antioxidants in dietary and skin care products has increased in popularity within the past several years. Aggressive investigations of RSV have been underway for the past twenty years [1]. Resveratrol is considered to be a very potent antioxidant, which makes it a unique candidate to provide desirable anti-aging effects in cosmetic products. Research has indicated that polyphenols found in wines are some of the most potent antioxidants, which are often several times more potent than vitamins A, C, and E [1]. Idebenone is considered to be the most potent topical antioxidant, but new studies have indicated that resveratrol has approximately 17-fold higher potency compared to idebenone [1]. The topical use of resveratrol appears to pose very few risks to consumers while having very large benefits, which allows formulators to target specific formulations to be marketed.

However, resveratrol has limitations as well. One big limitation is that the compound is not water soluble, and therefore, has to be dissolved using other means, such as organic solvents/oils, which are harmful to the environment and the human body. Additionally, resveratrol has stability issues, even in organic solvents. Therefore, increased water solubility and stability of resveratrol are needed for any commercially successful formulation.

Current drug delivery systems, such as polymers and lipids, all have their own restrictions that may include poor reproducibility, water solubility, loading capacity, and stability [8]. Moreover, often there is a necessity to combine molecules that are hydrophobic and hydrophilic within a single formulation, which can cause incompatibility issues and requires sophisticated solutions. There is a need for a multifunctional delivery system, such as dendrimers, that can solve more than one problem [9]. The objective of this research was to determine if the use of multifunctional dendrimers can enhance the solubility and stability of *trans*-resveratrol to prepare an aqueous topical cream formulation with enhanced RSV concentration. Additional goals included scaling-up any promising dendrimer-RSV formulation for commercial application.

We first reported (no data provided) the role of dendrimers to enhance the solubility and stability of RSV in a conference publication and we have also filed a patent on the proposed work (PCT/US2014/052105) [10]. In this research paper, we will examine the benefits of using dendrimers to enhance: (i) the solubility; (ii) the stability and (iii) the transdermal penetration of *trans*-resveratrol to develop an advanced topical anti-aging cream.

**Figure 2:**
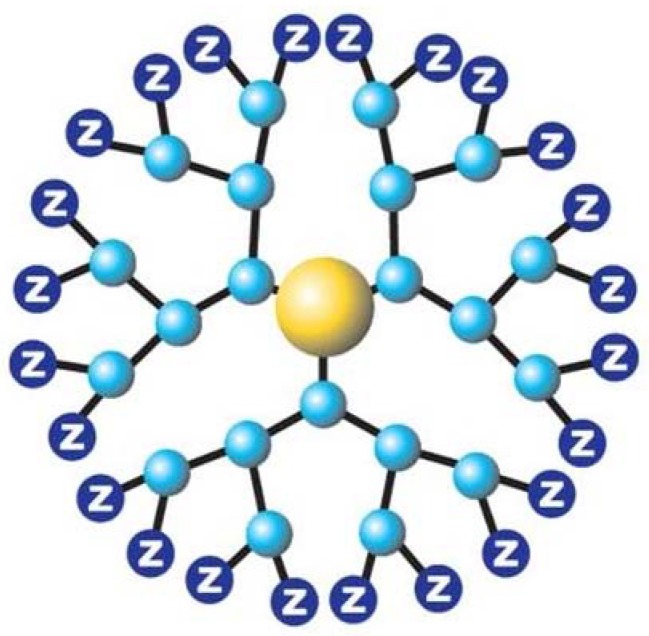
Dendrimer structure.

Dendrimers have unique spherical architecture and polyvalency at the nanometer range (Figure 2). The nanoscale structure of a dendrimer is made up of a main core that is extended out by generation after generation branching process around the central core and ends in polyvalent surface groups [11]. Dendrimer comes from the Greek word dendron, which means “tree” [10]. The dendrimer structure demonstrates the capacity to control size of the dendrimer based upon the number of generations, along with the surface functionality provided by using different surface groups. Dendrimer architecture can be tuned to get the desired size and surface to suit a particular drug entrapment problem. This entrapment in the dendrimer allows hydrophobic molecules to dissolve easily in water, and both hydrophobic and hydrophilic molecules can be entrapped together in a single dendrimer structure [12].

We have demonstrated that dendrimers can be used to enhance the water solubility of different drugs [10,11]. We have also shown that drug release can be controlled by physical and chemical modification of the dendrimer structure [11]. We propose in this work to use PAMAM generation 4 (PAMAM G4) to entrap resveratrol and thus enhance its solubility, stability and transdermal permeation. The resulting dendrimer-resveratrol formulation will be optimized and scaled-up to attain a commercially viable formulation.

## 2. Results and Discussion the sections of this paper are in the wrong order – Results and Discussion goes before the Experimental. Please double check.

### 2.1. RSV Calibration Curve

The RSV calibration curve was created by plotting the RSV concentration against peak area obtained by HPLC. The calibration equation used was: Y = 3.0733x + 0.0186 (R^2^ = 1).

### 2.2. Solubility Enhancement of RSV through Dendrimer Complexation: Protocol 1 vs. Protocol 2

**Figure 3:**
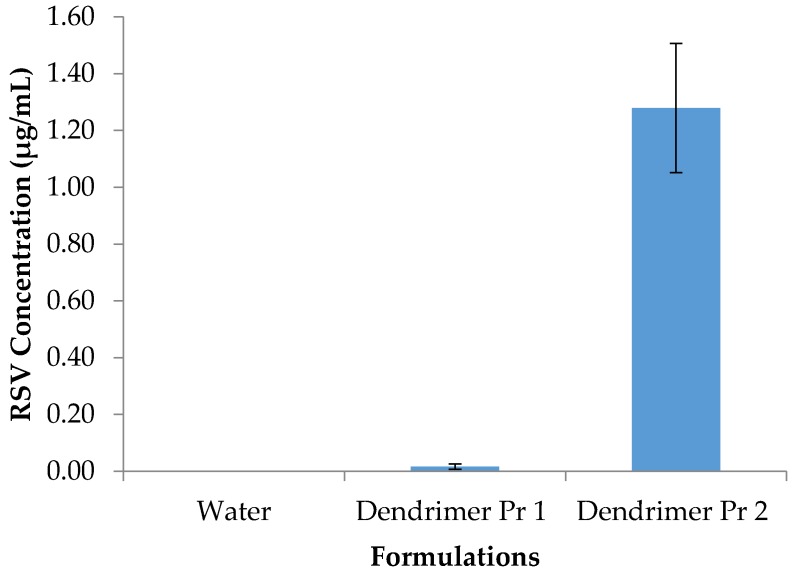
RSV solubility in water and with dendrimer formulations prepared by two different methods, Protocol 1 (Pr 1) and Protocol 2 (Pr 2). Dendrimer concentration 0.1% *w*/*v*. (*n* = 3, *p* < 0.05).

RSV entrapment in dendrimer aqueous solution was tested by two methods. In Protocol 1, RSV powder was exposed to aqueous dendrimer solution, but in Protocol 2, RSV was dissolved in methanol so that it would be available in the molecular form and then combined with dendrimer in the aqueous solution. In Protocol 2, all the solvents were removed by lyophilization and the final formulation was only in water. Protocol 1 and Protocol 2 were tested (Figure 3) at 0.1% *w*/*v* dendrimer concentration. RSV (control) in water did not show any solubility, but dendrimer formulations did show solubility enhancement of RSV in water. Dendrimer formulation showed 1.28 ± 0.23 µg/mL RSV concentration with Protocol 2 compared to 0.02 ± 0.01 µg/mL with Protocol 1. As Protocol 2 demonstrated significantly higher solubility enhancement propensity compared to Protocol 1 (Figure 3), it was decided to use Protocol 2 for further experimentation. RSV entrapment in dendrimer was mainly attributed to the electrostatic interactions, hydrogen bonding and molecular encapsulation as reported in our previous publications [10,13,14].

### 2.3. Optimization of Dendrimer Amount within the Formulation (Solubility Profile)

The experiment objective was to hold the amount of resveratrol added constant amongst the samples while varying the amount of dendrimer per sample in an attempt to find the optimum amount of dendrimer needed for the dendrimer-RSV formulation.

**Figure 4:**
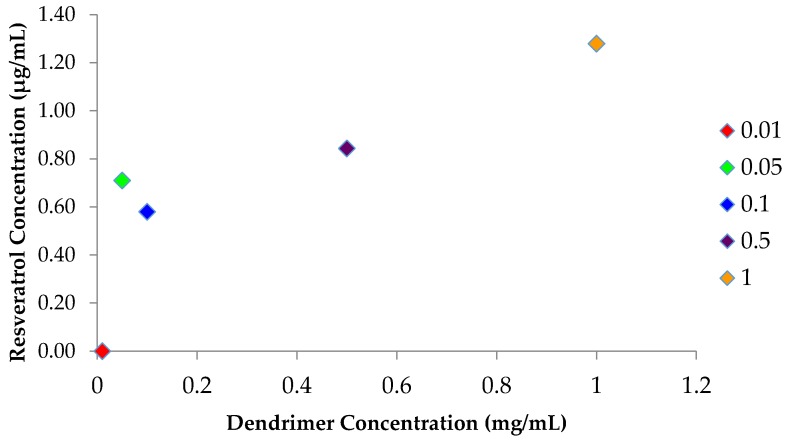
Solubility profile of dendrimer-RSV complex. Mean RSV concentration plotted against dendrimer concentration (*n* = 3), *p* < 0.05.

Figure 4 shows Higuchi’s AN type curve, where RSV concentration increases in a non-linear fashion with increasing dendrimer concentration. This pattern is similar to the pattern observed by our previous findings and can be attributed to nanoscale sterically induced stoichiometry (NSIS) [3,4,10,11,13,14]. The graph (Figure 4) also suggests that the 0.05 mg/mL concentration of dendrimer is the overall best concentration for entrapment potential (RSV/dendrimer mole ratio: 1:1).

### 2.4. Effect of pH on Dendrimer-RSV Complexation

Complexation of a drug in dendrimer depends upon the ionization state of both drug and dendrimer at a particular pH. RSV is a weak acid with hydroxyl groups having pKa values of 9.3, 10.0 and 10.6, respectively. Hence, RSV will be ionized under basic conditions and remain un-ionized under acidic conditions. On the contrary, dendrimers with basic amine surfaces will be ionized under acidic conditions and un-ionized under basic conditions. At pH 2.5, the dendrimer will be fully ionized but RSV will not be ionized and hence electrostatic complexation would not happen or by minimal. At pH 2.5, RSV has no solubility in water, but with 1% dendrimer, 0.216 µg/mL (0.0009 mM) RSV was observed (Table 1). This increase in solubility would be due to the molecular entrapment of RSV in dendrimer. Solubility of dendrimer-resveratrol at pH 2.5 was still lower than the solubility of dendrimer-resveratrol at pH 7. At pH 7.0, both dendrimer and RSV were partially ionized. When the resveratrol was added to the dendrimer (1%) formulation at pH 7.0, 2.65 µg/mL RSV concentration was achieved.

We tested the complex stability of RSV inside dendrimer by changing the ionic conditions after the dendrimer-RSV complex formation. When the pH of the dendrimer-RSV formulation (prepared at pH 7.0, 2.65 µg/mL) was reduced to pH 2.5, 1.09 µg/mL RSV was observed. Although RSV concentration reduced but it was still many fold better than the dendrimer-RSV complex prepared at pH 2.5 (0.216 µg/mL). Thus, the dendrimer protects the entrapped RSV from the extreme pH conditions, suggesting that the dendrimer can prevent resveratrol from attack under harsh acidic conditions, even those prevalent inside the gastro-intestinal tract.

### 2.5. Dissolution Studies

In the dissolution studies, the dendrimer-resveratrol formulation dissolved far more rapidly than resveratrol alone in both simulated gastric and simulated intestinal fluid. 

**Figure 5:**
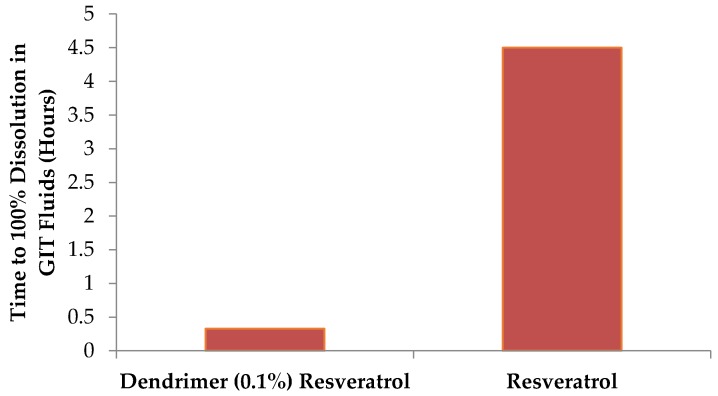
Time taken for RSV formulations to dissolve in GIT and intestinal fluids.

The dendrimer-resveratrol formulations reached 100% dissolution in 20 min in both the simulated gastric and simulated intestinal fluids (Figure 5), while resveratrol alone was still dissolving at the final time point in the experiment, 4.5 h. Hence dendrimer-resveratrol will dissolve very fast in gastro-intestinal tract and may provide higher bioavailability compared to the RSV alone.

### 2.6. Transdermal Permeation Study of Dendrimer-RSV Formulation

**Figure 6:**
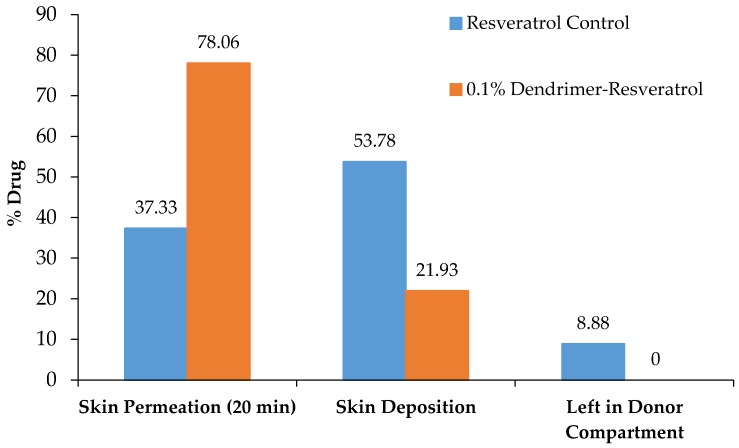
Transdermal Permeation of RSV formulations.

The dendrimer-resveratrol formulation showed 78.06% transdermal permeation compared to only 37.33% for resveratrol alone. Both formulations depicted maximum transdermal permeation in 20 min. Significantly higher quantities of RSV were found on the skin (53.78% vs. 21.9%) and the donor compartment (8.88% vs. 0%) for the resveratrol control formulation compared to the dendrimer-resveratrol formulations (Figure 6). This clearly indicates that dendrimer promotes resveratrol transdermal permeation into the skin. Furthermore, no dendrimer peak was observed by HPLC in the receptor compartment. This indicated that dendrimer did not cross the skin, but promoted the permeation of resveratrol across the skin. Dendrimer-RSV complex would penetrate deep into the skin compared to the RSV alone.

### 2.7. Resveratrol Stability Studies in Solution

A stability study of RSV was conducted at three temperatures as per the industry guidelines with RSV in methanol, RSV in Tween 80 and RSV in dendrimer. We have developed a HPLC method to observe the intact RSV and degraded RSV peaks in a single chromatogram. 

**Figure 7:**
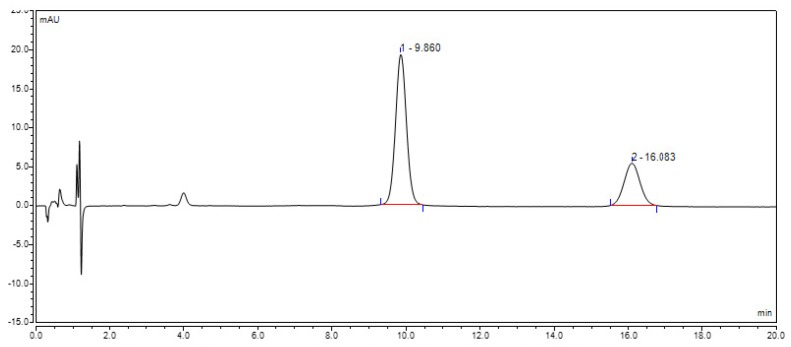
Chromatogram illustrating stable and degraded RSV peaks.

The HPLC chromatogram illustrated two peaks for resveratrol (Figure 7). One peak is shown at the retention time of 9.8 min, which corresponds to the stable resveratrol (*trans*-resveratrol). The second peak shown in the chromatogram at the retention time of 16.0 min, corresponds to the degraded resveratrol (*cis*-resveratrol).

**Figure 8:**
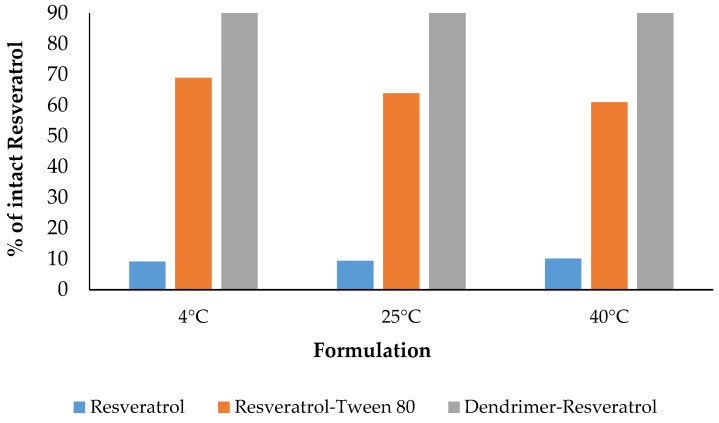
Percentage of intact resveratrol remaining in the formulations after Day 1.

All the formulations have 100% drug at day 0 and at Day 1. RSV + methanol lost almost all the drug. RSV + Tween 80 appears more stable than RSV + methanol, but less stable than RSV + dendrimer formulation (Figure 8). Dendrimer-RSV formulation remained significantly stable even after 60 days.

**Figure 9:**
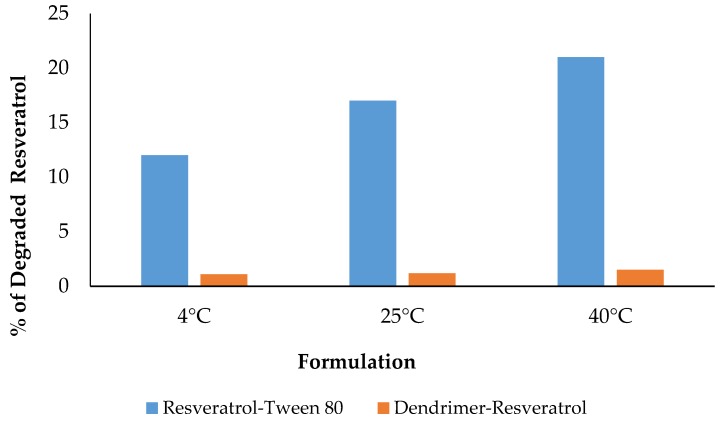
Percentage of degraded resveratrol remained in the formulations after Day 1.

We also compared the % of degraded RSV with time among the RSV formulations. We have found that dendrimer-RSV formulation showed negligible degraded RSV, whereas resveratrol + Tween 80 showed 12%–21% degraded RSV depending upon the storage temperatures (Figure 9). This pattern continued till day 60.

Enhanced stability of resveratrol was due to entrapment within the dendrimer nanostructure, thus preventing solvent exposure and minimizing degradation in aqueous solution.

### 2.8. RSV Cream Extraction Method Development and Testing of Commercial Products

An estimation method was developed to extract RSV from the semisolid formulations to analyze the amount of resveratrol. The extraction method developed was validated for accuracy and precision. The efficiency of the extraction method was 94% ± 4.5%. This extraction method was then used to analyze RSV in the commercially available RSV formulations/products.

**Figure 10:**
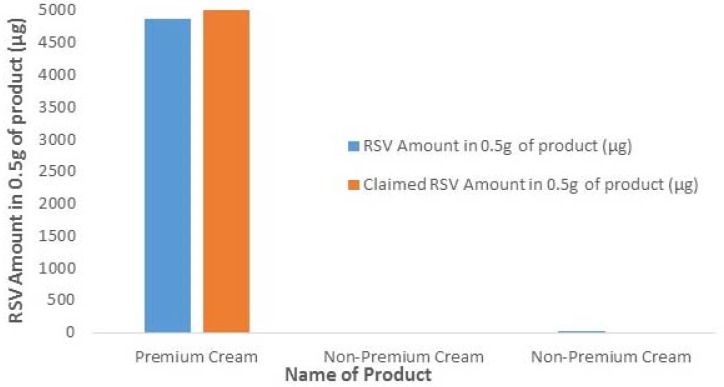
Amount of RSV extracted from the commercial products.

Figure 10 shows the testing of three commercially available resveratrol products. One product consisted of a premium market RSV product (only available online) and the other two were non-premium market resveratrol products. A premium cream is considered one that contains pure *trans*-resveratrol, which in this case contained 1% pure *trans*-resveratrol. A non-premium cream is defined as a cream that contains RSV extract and may not contain any pure *trans*-resveratrol form. The premium product had significant amounts of resveratrol in the product compared to the remaining products tested. The two non-premium products did not disclose the amount of resveratrol contained within their product and were protected under intellectual property rights. These non-premium products showed little to no RSV at all, which was rather interesting due to the fact their ingredients list included RSV. Overall, this data validates the developed RSV extraction protocol for testing RSV in the commercial products. Results also indicate that the amount of RSV in commercial products may be either less than claimed or they may even be devoid of any RSV. This could be due to the stability issues of RSV and need more study of RSV-based products.

### 2.9. Stability Study with Cream Formulation

**Figure 11:**
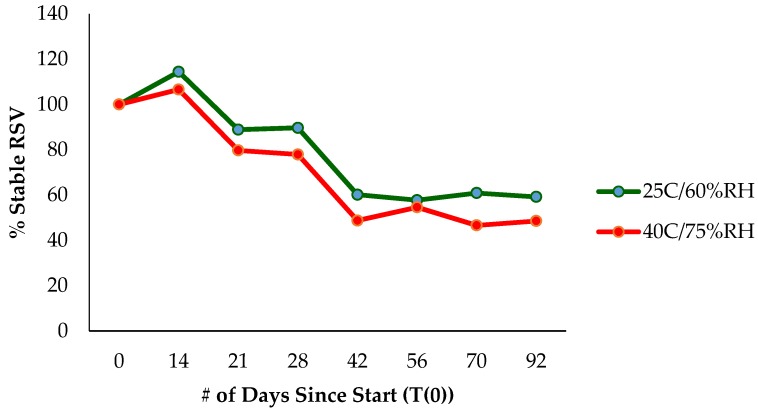
Cream stability studies, illustrating amount of stable resveratrol remaining.

**Figure 12:**
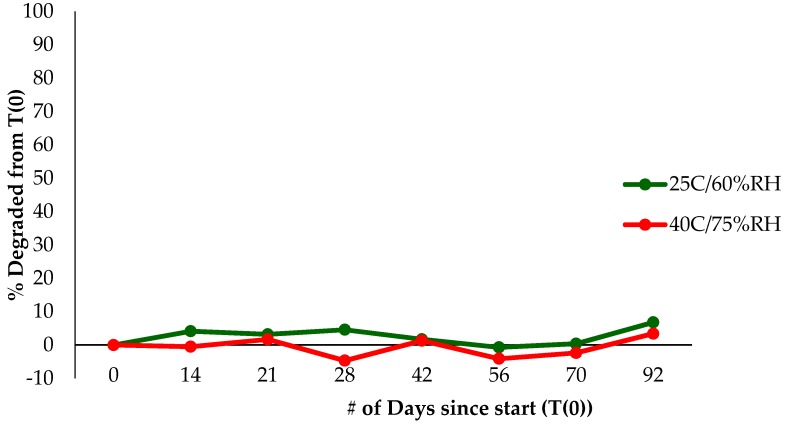
Cream stability studies, illustrating amount of degraded resveratrol over time in % degraded from starting time point.

Figure 11 and Figure 12 show the results of a 3-month stability study of the cream samples held at 25 °C/60% RH and 40 °C/75% RH. The results indicate that the dendrimer is able to hold resveratrol well, even at 92 days. Although approximately 50% had been lost over 92 days compared to the resveratrol alone in cream, this is considerably better than the stability of only ~56 days of RSV in solution under accelerated temperature. However, the degraded resveratrol graph signifies a very small to no increase in degraded RSV over the 92 days. Approximately only a >5% increase in degraded RSV was seen over the 92-day study. This shows that dendrimer was able to halt the degradation of resveratrol along with increasing its solubility.

### 2.10. Formulation Optimization and Scale-Up of Resveratrol + Dendrimer Formulation

#### 2.10.1. Optimization of Excess Resveratrol Added

**Figure 13:**
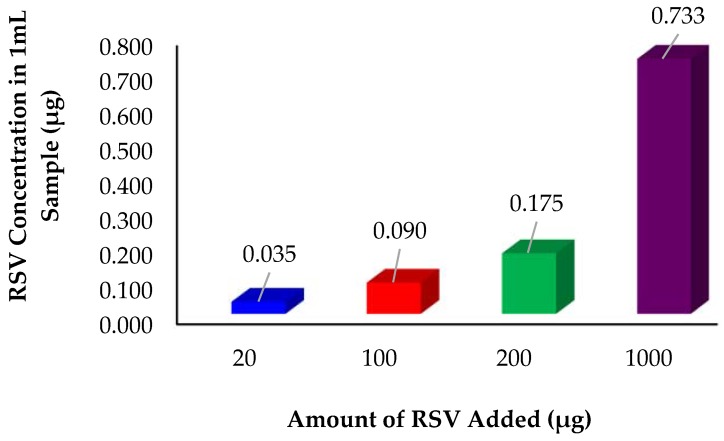
Reduction of resveratrol addition in excess in an attempt to save on overall drug required to hit target concentration. Mean RSV concentration (*n* = 3), *p* < 0.05.

The purpose was to reduce the amount of resveratrol added in excess from the original amount of 5 mg used for initial formulation development. Figure 13 illustrates that decreasing the excess RSV resulted in decrease in RSV loaded into the dendrimers. The amount of 0.73 μg RSV loading with 1000 μg RSV added is far higher than the 200 μg, 100 μg and 20 μg samples. Overall the amount of resveratrol added in excess needs to remain high to maintain equilibrium and ensure proper loading of the dendrimers to achieve the highest amount of RSV possible.

#### 2.10.2. Recovery of Excess Resveratrol from Syringe Filters for Reuse

**Figure 14:**
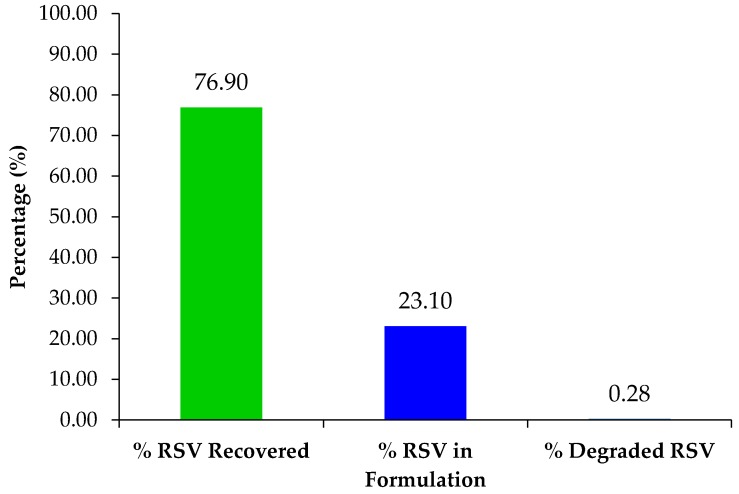
Percent RSV recovered from syringe filters, formulation and degraded. Mean RSV concentration (*n* = 3), *p* < 0.05.

The overall purpose of this experiment was to extract the remaining RSV in excess from the syringe filters to illustrate that the excess RSV could be recovered and reused in further studies if necessary. 23.1% RSV was used up in the formulation and remaining 76.9% RSV could be retrieved for reuse (Figure 14). This means that resveratrol would not be wasted and helps to reduce overall costs in formulation scale up.

#### 2.10.3. Preparation of Concentrated Dendrimer-RSV Solution

**Figure 15:**
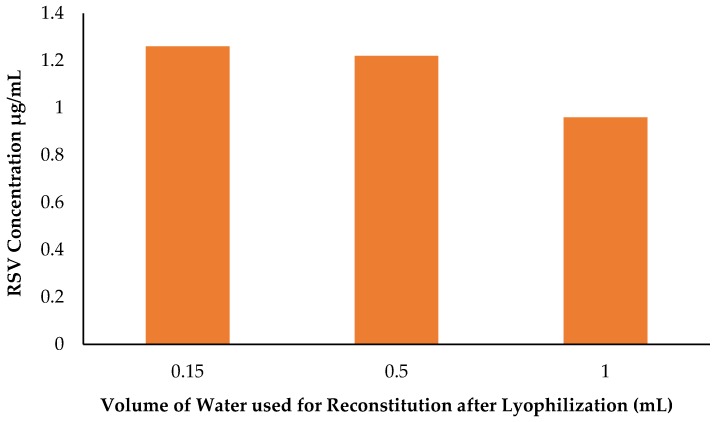
Reduction of reconstitution volume of H_2_O added after lyophilization (mL).

The purpose for this experiment was to explore if concentrated dendrimer-RSV solution can be prepared for commercial applications without losing RSV. Figure 15 clearly illustrates that the volume of reconstitution can be reduced to prepare a concentrated dendrimer-RSV formulation. Originally the RSV concentration was 0.96 μg/mL using 1 mL water for reconstitution and it was found to be 1.22 μg/mL and 1.26 μg/mL with 0.5 and 0.15 mL water for reconstitution, respectively. This data also indicates that RSV is entrapped inside the dendrimer and hence reduction in reconstitution volume is not affecting the RSV loading.

#### 2.10.4. Scale up of Dendrimer−Resveratrol formulations

**Figure 16:**
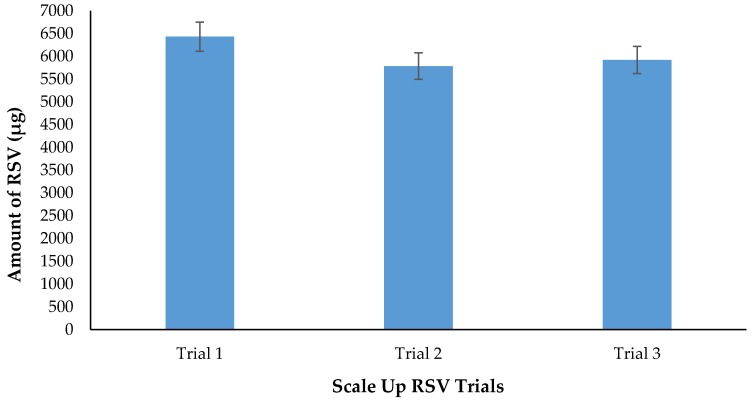
Dendrimer- RSV scale up batches. Mean RSV concentration (*n* = 3), *p* < 0.05.

The purpose of this experiment was to scale up the small bench scale to a larger commercial scale. We have successfully prepared a 100 mL dendrimer-RSV formulation, validated for reproducibility through multiple trials (Figure 16). Therefore, it has been determined that the method is robust and can be used to make further scale up batches.

## 3. Materials and Methods

### 3.1. Materials 

The materials used for this formulation include methanol, cyclohexane, water, resveratrol and dendrimers. The methanol came from OmniSolv (Billerica, MA, USA). The cyclohexane was manufactured by Honeywell Burdick & Jackson (Muskegon, MI, USA). The water used was from a Millipore system using a 0.18 μm filter. Generation 4, diaminobutane core, amine surface, poly(amidoamine) (PAMAM) dendrimers were purchased from NanoSynthons (Mount Pleasant, MI, USA) and *trans*-resveratrol from Sigma-Aldrich (Milwaukee, WI, USA).

### 3.2. Methodology

#### 3.2.1. Calibration Curve of RSV by HPLC

The calibration graph of RSV was prepared in methanol using HPLC. The standard samples were analyzed via a Dionex UltiMate 3000 HPLC (Thermo Scientific, Waltham, MA, USA) using a Luna C18 column (5 μ 100 A Size: 100 × 4.6 mm, Phenomenex, Torrance, CA, USA). Chromeleon software was used to control the UltiMate 3000 and analyze the data generated. The system flow rate was set to 1.0 mL/min and mobile phase ratio was isocratic throughout the run at a 40% methanol: 60% water for a 20-min run period. The diode array detector of the Ultimate 3000 HPLC was set to a wavelength of 308 nm. Injection volumes for the samples were 25.00 µL per sample.

#### 3.2.2. pH Modification of PAMAM Dendrimer

pH of the PAMAM dendrimer was modified to the physiological pH 7.0 and 2.5 by 1 N HCl.

#### 3.2.3. Dendrimer-Resveratrol Formulation (Solubility Enhancement)

Two different entrapment protocols were developed, namely Protocol 1 and Protocol 2. Control samples were prepared by adding solid RSV in water.

##### Protocol 1: Entrapment process in water alone.

RSV in excess (2 mg) was added to amber vials containing a total volume of 1 mL, made up of a combination of dendrimer (1 mg, 33.0 µL) and Millipore water (967.0 µL). Each vial was sonicated for 15 s in three 5-s intervals, and then placed in an orbital water bath shaker at 37 °C, protected from light using aluminum foil and shaken overnight. The suspensions were filtered through a 13 mm 0.2 µm nylon syringe filter. One hundred μL of formulation was then diluted with HPLC-grade methanol in an amber HPLC vial and analyzed by HPLC for RSV concentration.

##### Protocol 2: Entrapment process in water + organic solvent mixture.

RSV in excess (2.0 mg) was added to amber vials containing methanol (500 µL) and vortexed for 15 s. RSV in methanol was then transferred into new amber vials containing dendrimer (1 mg, 33.0 µL) and Millipore water (467 µL). Each vial was sonicated for 15 s in three 5-s intervals and placed in an orbital water bath shaker and shaken at 37 °C, protected from light using aluminum foil for at least 2 h. Upon completion of shaking, the vials underwent lyophilization overnight to remove water and methanol, and were reconstituted with Millipore water (1000 µL). The formulations were placed in an orbital water bath shaker (37 °C), protected from light using aluminum foil and shaken for 2 h. The suspensions were filtered through a 13 mm 0.2 µm nylon syringe filter. One hundred μL of formulation was then diluted with HPLC-grade methanol in amber HPLC vial and analyzed by HPLC for RSV concentration.

On the basis of better RSV solubility enhancement, Protocol 2 was be used for further experimentations.

### 3.3. Optimization of Dendrimer Amount and Preparation of the Solubility Profile

After establishing methods to entrap RSV in dendrimer, the efficacy of formulation was evaluated by reducing the amount of dendrimer from the original amount of 1.0 mg used to entrap RSV. The purpose was to vary the concentrations of dendrimer, while holding the concentration of added RSV constant. The following dendrimer concentrations were tested using Protocol 2: 0.01, 0.05, 0.1 0.5 and 1 mg/mL. The solubility profile (Figure 4) was created by plotting dendrimer concentration against RSV concentration in the final formulation.

### 3.4. Effect of pH on Dendrimer-RSV Complexation

An examination of the effect of pH on the solubility of RSV in the dendrimer formulations was approached in two ways. Two dendrimer solutions (0.1%) were prepared according to Protocol 2. In the first set of formulations, dendrimer solution was first modified to pH 2.5 by 1 N HCl and then RSV was added to prepare dendrimer-RSV complex. In the second set of formulations, dendrimer-resveratrol complex was first formed using pH 7.0 dendrimer (Section 3.3, Protocol 2), and then pH of this final formulation was lowered pH 2.5. Additionally, a control formulation of RSV in pH 2.5 water was prepared.

### 3.5. Dissolution Studies

An examination was made regarding dissolution of RSV in simulated gastric and intestinal environments. Simulated gastric and intestinal fluids were prepared, with the simulated gastric fluid consisting of HCl, NaCl, Pepsin, and water while the simulated intestinal fluid consisted of NaOH, KH_2_PO_4_, and water. 1 mL of a 0.1% dendrimer-resveratrol formulation was prepared using Protocol 2 and analyzed via HPLC. The formulation was split into two equal aliquots and lyophilized. Ten mL of the simulated solutions were added to the respective lyophilized dendrimer-resveratrol formulation, and the fluids were stirred at a low speed (1 on an arbitrary 10-point scale stir plate). This experiment was protected from light. At various time points, 0.5 mL aliquots of fluid were removed from the chambers and analyzed via HPLC. The volume removed was replenished with fresh simulated fluid. Control formulations of resveratrol alone, were analyzed simultaneously and compared to the dendrimer-containing formulations.

### 3.6. Transdermal Permeation Study

Transdermal permeation studies were conducted using Franz Diffusion Cells (FDC) and rat skin samples. The diffusion cells had a 5 mL receptor chamber and were manufactured by Permegear (Hellertown, PA, USA). The formulations examined were a 0.1% dendrimer-resveratrol formulation, and a control of free resveratrol in water. For each formulation, the receptor chamber of the FDC was filled with 5 mL of a PBS (pH 7.4):methanol 90:10 mixture. Skin samples from Dahl Salt Sensitive rats were cut to size and placed at the interface of the donor and receptor chambers. 0.5 mL of the desired formulation was placed in the donor chamber and the FDC was stirred on a stir plate set to 6 (on an arbitrary 10-point scale). At various intervals over a 24-h period, 400 μL was removed from the receptor chamber by means of the sampling arm and analyzed by HPLC. The volume was replaced by an equal amount of fresh PBS: methanol mixture. After 24 h, an aliquot was taken from the donor chamber and the skin was removed and RSV recovered by soaking skin in 2 mL methanol and sonicating for 10 min followed by filtration and HPLC analysis as discussed before.

### 3.7. Stability Study of Resveratrol, Resveratrol + Tween 80 and Resveratrol + Dendrimer in Aqueous Solution

Purpose of the stability testing of RSV in solution was to determine how long RSV is stable in aqueous solution to predict its shelf life expectations. RSV formulations with dendrimer and Tween-80 were prepared along with a control in methanol. Samples were stored at Wisconsin Pharmacal Company (WPC, Jackson, WI, USA) under ICH Q1A conditions in the following temperatures: 4 °C/0% RH, 25 °C ± 2 °C/60% ± 5% RH, 40 °C ± 2 °C/75% ± 5% RH. The samples were collected on the following time points: Day 0, 1, 3, 7, 10, 14, 21, 1 month, and 2 months. The collected samples were measured for RSV content by HPLC. A graph was plotted between percent remaining RSV against time for each temperature point to evaluate stability of the formulations.

### 3.8. Resveratrol + Dendrimer in Cream Dosage Form

#### 3.8.1. Method Development for Extraction of RSV from Cream Formulations (RSV Cream Extraction Method)

The purpose was to develop a method to extract RSV from the cream formulations in order to analyze the RSV content. RSV (1 mg) was dissolved in methanol (1 mL). This RSV solution was then mixed with cream (0.5 g, provided by WPC) in a 30 mL amber glass vial and properly mixed to prepare a homogeneous formulation. Next, methanol (9 mL) was added to the cream which was vortexed. Cream was warmed in a water bath at 37 °C for 5 min and placed in an ice bath for 15 min. The cream mixture was subjected to the gravity filtration (W&R Balston Filter Paper No. 3, 15 cm) into a 15 mL plastic conical vial. The filtered solution was centrifuged at 4000 rpm in a 5810 centrifuge (Eppendorf, Hauppauge, NY, USA) for 15 min. After the sample was centrifuged, the supernatant was decanted into a 125 mL separatory funnel. The methanol based mixture was treated with cyclohexane (7 mL). Six cyclohexane extractions were performed. After all the extraction steps, the collected methanol fractions were combined and filtered using a 13 mm 0.2 μm syringe filter into a new 30 mL amber glass vial. The sample underwent HPLC analysis.

#### 3.8.2. Stability Study of Resveratrol + Dendrimer in Cream Formulation

Purpose for this experiment was to test the stability of the resveratrol-dendrimer complex in finished cream formulation by conducting a 3-month stability study. Dendrimer-RSV formulation was mixed using a drill mixer at slow rpms to ensure even distribution of the formulation. Using a 20 mL syringe, cream (1 g, provided by WPC)) was added to each product container and the plunger within the tube was pushed up to remove excess air. Formulations were placed under ICH Q1A conditions of: 25 °C ± 2 °C/60% ± 5% RH, 40 °C ± 2 °C/75% ± 5% RH. Samples were collected at the following intervals: Day 0, 1, 2, 3, 4, 6 and 8 weeks. Stability graph was plotted between % remaining RSV against time for each temperature point.

#### 3.8.3. Extraction of Resveratrol from Commercially Available Resveratrol Products

The purpose for conducting this experiment was to test commercial RSV products to see if the claimed amount of resveratrol was present in the products. Each product (0.5 g) was subjected to the developed cream extraction protocol and the RSV content estimated.

### 3.9. Formulation Optimization and Scale-Up of Resveratrol + Dendrimer Formulation

#### 3.9.1. Reduction of Excess RSV Added to Initial Stock Formulation

The purpose was to reduce amount of RSV added to the initial formulation to reduce overall formulation costs. The remaining steps were followed according to Protocol 2.

#### 3.9.2. Reduction of Reconstitution Water Volume Post Lyophilization

The purpose was to reduce the reconstitution volume required to prepare concentrate dendrimer-resveratrol formulation. Reconstitution volume was reduced from 1 mL to 0.15, 0.5 and 0.1 mL. The remaining steps were followed according to Protocol 2.

#### 3.9.3. Extraction of Excess Resveratrol from Syringe Filters for Reuse

The overall purpose of this experiment was to extract the remaining uncomplexed RSV from the syringe filters to illustrate that the excess RSV could be recovered and reused in further studies if necessary. The syringe filter(s) were placed into a 250 mL media bottle and soaked in approximately 100 mL of fresh methanol. The filters were swirled well and analyzed by HPLC.

### 3.10. Formulation Scale-Up

The purpose for this experiment was to scale up the RSV+ dendrimer formulation from 1 mL to 100 mL formulation. Also we aimed to increase the RSV concentration from 1 μg/mL to 10 μg/mL. First RSV (100 mg) was added to a 500 mL media bottle followed by the addition of fresh methanol (50 mL) and stirred well. Next, in a separate 500 mL media bottle, dendrimer (4.18 mL, 5 mg or 0.005% *w*/*v*) was added along with Millipore (Billerica, MA, USA) H_2_O (45.82 mL) and mixed well. The two solutions were mixed together in the media bottle containing the RSV. The solution was then sonicated for 5 min at sixty 5 s intervals. After sonication, the media bottle was covered with aluminum foil and placed in a 37 °C orbital shaker for 2 h. Upon completion of stirring, the solution was then lyophilized overnight. Post-lyophilization, the formulation was reconstituted using Millipore H_2_O (100 mL) and mixed well. The formulation was then sonicated for 5 min in 60 5 s intervals. After sonication, the formulation was placed into the 37 °C orbital shaker for 2 h. Then the formulation was removed from the shaker and filtered using a 13 mm 0.2 μm nylon syringe filter into a fresh 500 mL media bottle. The filtered solution was then analyzed by HPLC.

## 4. Conclusions

We have demonstrated that the solubility, stability and transdermal permeation of resveratrol can be enhanced by entrapment in the dendrimer architecture. RSV entrapment in dendrimer is due to electrostatic interactions, hydrogen bonding and molecular encapsulation. Entrapment of the resveratrol within the dendrimer helps prevent the resveratrol from being exposed to the solvent and reduces the overall degradation within an aqueous solution [14]. Since the dendrimer-resveratrol complex is an aqueous formulation, it does not need any additional excipients that are typically used within the cosmetic industry. This aqueous formulation can be simply mixed into any formulation and at any stage in the development of a product. Another benefit of the dendrimer-resveratrol complex is that the process is very neat and does not need force or heat, unlike other drug delivery systems [14].

We have successfully optimized and scaled up the dendrimer-RSV formulation. Overall the results obtained have produced a finished product that could be placed into a topical anti-aging cream or into other customers’ products for various applications. Additionally, the results obtained have indicated that the concentration of the overall finish product (~50 μg/mL) is roughly five times higher than that of many premium commercial products, which contain 1% resveratrol (~10 μg/mL). The use of the dendrimers with resveratrol also increases the stability of the product, which therefore would help in keeping the product in a useable condition for a customer for a longer period of time. Dendrimer usage also allowed for creating the product that was mainly water-based compared to other products which contain harsh organic solvents that are harmful or toxic to the body. Clinicaltrials.gov showed 117 studies for resveratrol and this indicates a need for an advanced formulation to effectively deliver RSV. We can safely claim to make a water based, stable, ‘green’ nanoformulation of resveratrol with enhanced skin penetration, which is scaled-up and ready to use for the commercial applications. We also envision the use of aqueous dendrimer-RSV complex for intracellular RSV delivery for cell line mechanistic studies.

## Figures and Tables

**Table 1:** Effect of pH on solubility of resveratrol and dendrimer-resveratrol complex.

Formulations	pH 2.5	pH 7.0
Resveratrol	zero	0.015 µg/mL
Dendrimer-resveratrol	0.216 µg/mL	2.65 µg/mL
